# A new inequality for the Riemann-Stieltjes integrals driven by irregular signals in Banach spaces

**DOI:** 10.1186/s13660-018-1611-4

**Published:** 2018-01-15

**Authors:** Rafał M Łochowski

**Affiliations:** 1 Department of Mathematics and Mathematical Economics, Warsaw School of Economics, ul. Madalińskiego 6/8, Warsaw, 02-513 Poland; 2 African Institute for Mathematical Sciences, 6-8 Melrose Road, Muizenberg, 7945 South Africa

**Keywords:** 46B99, 46G10, regulated path, total variation, *p*-variation, truncated variation, the Riemann-Stieltjes integral, the Loéve-Young inequality, Banach space

## Abstract

We prove an inequality of the Loéve-Young type for the Riemann-Stieltjes integrals driven by irregular signals attaining their values in Banach spaces, and, as a result, we derive a new theorem on the existence of the Riemann-Stieltjes integrals driven by such signals. Also, for any $p\ge1$, we introduce the space of regulated signals $f:[a,b]\rightarrow W$ ($a< b$ are real numbers, and *W* is a Banach space) that may be uniformly approximated with accuracy $\delta>0$ by signals whose total variation is of order $\delta^{1-p}$ as $\delta\rightarrow0+$ and prove that they satisfy the assumptions of the theorem. Finally, we derive more exact, rate-independent characterisations of the irregularity of the integrals driven by such signals.

## Introduction

The first aim of this paper is a generalisation of the results of [1] and [2] to the functions attaining their values not only in $\mathbb{R}$ but in more general spaces. Next, to obtain more precise results, for any $p\ge1$, we introduce the space $\mathcal{U}^{p} ( [a,b ], W )$ of regulated functions/signals $f:[a,b]\rightarrow W$ ($a< b$ are real numbers, and *W* is a Banach space) that may be uniformly approximated with accuracy $\delta>0$ by functions whose total variation is of order $\delta^{1-p}$ as $\delta \rightarrow 0+$. This way we will obtain a result about the existence of the limit of Riemann-Stieltjes sums, which we will denote by $\int_{a}^{b} f \,\mathrm{d}g$, for functions from $\mathcal{U}^{p} ( [a,b ] )$ and $\mathcal{U}^{q} ( [a,b ] )$ whenever $p,q>1$, $p^{-1}+q^{-1}>1$. Results of this type were earlier obtained by Young [3, 4] and D’yačkov [5] (for very detailed account, see [6, Chapter 3]), but they were expressed in terms of *p*- or (more general) *ϕ*-variations. The integral obtained as the limit of Riemann-Stieltjes sums in this case is often called the Young integral. It is in place to mention that nowadays there exists fairly rich literature on the integration with respect to irregular integrators where the object defined as an integral is not necessarily obtained as the limit of Riemann-Stieltjes sums. In [7] an interesting approach based on fractional calculus was developed. In the modern theory of rough paths developed by Terry Lyons, the existence of integrals of the form $\int_{0}^{t} f(y_{s}) \cdot\mathrm{d}x_{s}$ and *y* satisfying $y(t) = y(0) + \int_{0}^{t} f(y_{s}) \cdot\mathrm{d}x_{s}$ is proven, even for very irregular *x*, as long as *f* is sufficiently regular and one is provided with the values of the iterated integrals $\int_{t_{1}< s < t_{2}} \mathrm{d}x_{s}^{i}$, $\int_{t_{1}< s < t < t_{2}} \mathrm{d}x_{s}^{i}\, \mathrm {d}x_{t}^{j}$, $\int _{t_{1}< s < t <u < t_{2}} \mathrm{d}x_{s}^{i} \,\mathrm{d}x_{t}^{j}\, \mathrm{d}x_{u}^{k}$, *etc*.; see [8, 9]. Both approaches mentioned provide tools to deal also with the case where the integrand and integrator reveal the same irregularity as the standard Brownian motion or more general semimartingales (although the Young integral fails to exist in such case, and it was, historically, one of the main reasons for the development of the stochastic integral). To deal with the integrals driven by a fractional Brownian motion, which fails to be a semimartingale except the special case when its Hurst parameter is $1/2$, one uses various integrals: Young’s integral, fractional integral or the Skorohod integral; see [10, 11].

To obtain the convergence of Riemann-Stieltjes sums, we will use a partial solution of a variational problem similar to that considered in [1]. In [1] the following problem was considered: given real $a< b$, $c>0$, a regulated function/signal $f:[a,b]\rightarrow \mathbb{R}$ (for the definition of a regulated function see the next section) and $x\in[f (a )-c/2,f (a )+c/2 ]$, find the infimum of total variations of all functions $f^{c,x}:[a,b]\rightarrow \mathbb{R}$ that uniformly approximate *f* with accuracy $c/2$,

$$\bigl\Vert f-f^{c,x} \bigr\Vert _{[a,b],\infty }:=\sup _{a\le t\le b} \big\vert f (t )-f^{c,x} (t ) \big\vert \le c/2, $$

and start from $f^{c,x} (a )=x$. Recall that the total variation of $g:[a,b]\rightarrow \mathbb{R}$ is defined as

$$\operatorname{TV}\bigl(g,[a,b]\bigr) :=\sup_{n}\sup _{a\le t_{0}< t_{1}< \cdots< t_{n}\le b}\sum_{i=1}^{n} \big\vert g (t_{i} )-g (t_{i-1} ) \big\vert . $$

This infimum is well approximated by the *truncated variation* of *f*, defined as

1 $$ \operatorname{TV}^{c}\bigl(f,[a,b]\bigr) :=\sup_{n}\sup _{a\le t_{0}< t_{1}< \cdots< t_{n}\le b} \sum_{i=1}^{n} \max \bigl\{ \big\vert f (t_{i} )-f (t_{i-1} ) \big\vert -c,0 \bigr\} , $$

and the following bounds hold:

$$\operatorname{TV}^{c}\bigl(f,[a,b]\bigr) \le\inf_{f^{c,x}\in B (f,c/2 ),f^{c,x} (a )=x} \operatorname{TV}\bigl(f^{c,x},[a,b]\bigr) \le \operatorname{TV}^{c} \bigl(f,[a,b]\bigr) +c, $$

where $B (f,c/2 ):= \{ g: \Vert f-g \Vert _{[a,b] ,\infty }\le c/2 \} $ (see [1, Thm. 4 and Rem. 15]). Moreover, we have

2 $$ \inf_{f^{c}\in B (f,c/2 )} \operatorname{TV}\bigl(f^{c},[a,b]\bigr) = \operatorname{TV}^{c}\bigl(f,[a,b]\bigr) . $$

Unfortunately, this result is no more valid for functions attaining their values in more general metric spaces.

### Remark 1

It is not difficult to see that (2) does not hold even for *f* attaining its values in $\mathbb{R}^{2}$ with $|\cdot|$ understood as the Euclidean norm in $\mathbb{R}^{2}$. Indeed, let $f: [0,2 ]\rightarrow \mathbb{R}^{2}$ be defined by $f (t )= (\cos(2\pi\lfloor t \rfloor /3 ),\sin(2\pi\lfloor t \rfloor/3 ) )$. We have $\operatorname{TV}^{\sqrt{3}}(f,[0,2]) =0$, but there exists no sequence of functions $f_{n}: [0,2 ]\rightarrow \mathbb{R}^{2}$, $n=1,2,\ldots$ , such that $\Vert f-f_{n} \Vert _{[0,2],\infty }\le\sqrt{3}/2$ and $\lim_{n\rightarrow +\infty } \operatorname{TV}(f_{n},[0,2]) =0$. Thus for $c = \sqrt{3}$, $\inf_{f^{c}\in B (f,c/2 )} \operatorname{TV}(f^{c},[a,b]) > \operatorname{TV}^{c}(f,[a,b]) $.

Remark 1 answers (negatively) the question posed few years ago by Krzysztof Oleszkiewicz if the truncated variation is the greatest lower bound for the total variation of functions from $B(f,c/2)$ attaining values in $\mathbb{R}^{d}$, $d=2,3,\ldots$ , or in other spaces than $\mathbb{R}$. Fortunately, it is possible to state an easy estimate of the left side of (2) in terms of the truncated variation of *f*, for *f* attaining values in *any* metric space (to define the total variation and the truncated variation of *f* attaining its values in the metric space $(E,d )$, we just replace $|f (t_{i} )-f (t_{i-1} )|$ by the distance $d (f (t_{i} ),f (t_{i-1} ) )$); see Theorem 1.

One application of Theorem 1 will be a generalisation of the results of [2] on the existence of the Riemann-Stieltjes integral. We will consider the case where the integrand and integrator attain their values in Banach spaces. The restriction to the Banach spaces stems from the fact that the method of our proof requires multiple application of summation by parts and proceeding to the limit of a Cauchy sequence, which may be done in a straightforward way in any Banach space. This way we will obtain a general theorem on the existence of the Riemann-Stieltjes integral along a path in some Banach space $(E, \Vert \cdot \Vert _{E} )$ (with the integrand being a path in the space $L (E,V )$ of continuous linear mappings $F:E\rightarrow V$, where *V* is another Banach space) and an improved version of the Loéve-Young inequality for integrals driven by irregular paths in this space.

The famous Loéve-Young inequality may be stated as follows. If $f:[a,b]\rightarrow L (E,V )$ and $g:[a,b]\rightarrow E$ are two regulated functions with no common points of discontinuity and *f* and *g* have finite *p*- and *q*-variations, respectively, where $p > 1$, $q > 1$ and $p^{-1}+q^{-1}>1$, then the Riemann-Stieltjes integral $\int_{a}^{b}f\,\mathrm{d}g$ exists, and we have the following estimate:

3 $$ \biggl\Vert \int_{a}^{b}f\,\mathrm{d}g-f (a ) \bigl[g (b )-g (a ) \bigr] \biggr\Vert \le\tilde{C}_{p,q} \bigl(V^{p} \bigl(f,[a,b] \bigr) \bigr)^{1/p} \bigl(V^{q} \bigl(g,[a,b] \bigr) \bigr)^{1/q}. $$

Here

$$V^{p} \bigl(f,[a,b] \bigr):=\sup_{n}\sup _{a\le t_{0}< t_{1}< \cdots < t_{n}\le b}\sum_{i=1}^{n} \bigl\Vert f (t_{i} )-f (t_{i-1} ) \bigr\Vert _{L (E,V )}^{p} $$

and

$$V^{q} \bigl(g,[a,b] \bigr):=\sup_{n}\sup _{a\le t_{0}< t_{1}< \cdots < t_{n}\le b}\sum_{i=1}^{n} \bigl\Vert g (t_{i} )-g (t_{i-1} ) \bigr\Vert _{E}^{q} $$

denote the *p*- and *q*-variations of *f* and *g*, respectively (sometimes called the *strong* variations). The original Loéve-Young estimate with the constant $\tilde {C}_{p,q}=1+\zeta(1/p+1/q )$, where *ζ* is the famous Riemann zeta function, was formulated for real functions in [3]. The counterpart of this inequality for more general, Banach space-valued functions, with the constant $\tilde{C}_{p,q}=4^{1/p+1/q}\zeta(1/p+1/q )$, is formulated in the proof of [12, Theorem 1.16]. Our improved version of (3) is the following:

4 $$\begin{aligned}[b] & \biggl\Vert \int_{a}^{b}f\,\mathrm{d}g-f (a ) \bigl[g (b )-g (a ) \bigr] \biggr\Vert \\ & \quad\le C_{p,q} \bigl(V^{p} \bigl(f,[a,b] \bigr) \bigr)^{1-1/q} \Vert f \Vert _{\text{osc},[a,b]}^{1+p/q-p} \bigl(V^{q} \bigl(g,[a,b] \bigr) \bigr)^{1/q}, \end{aligned} $$

where $\Vert f \Vert _{\text{osc},[a,b]}:=\sup_{a\le s< t\le b} \Vert f (s )-f (t ) \Vert _{L (E,V )}$, and $C_{p,q}$ is a universal constant depending on *p* and *q* only. Notice that always

5 $$ \bigl(V^{p} \bigl(f,[a,b] \bigr) \bigr)^{1/p- (1-1/q )} \ge \Vert f \Vert _{\text{osc},[a,b]}^{1+p/q-p}. $$

Let us comment shortly on the proofs of (3) and related results that have appeared so far. Young’s original proof of (3) utlilised elementary but clever induction argument for finite sequences. Since then, there appeared several generalizations of (3), for example, based on control functions; see [8, Section 3.3] or [12, Section 1.3]. Another proof based on control functions but with different constant may be found in [13, Chapter 6]. An abstract version of the inequality proven in [13], called the *sewing lemma*, was proven in [14]. All these approaches give an inequality where only *p*- and *q*-variation (or Hölder) norms appear, whereas our approach gives an estimate where the *p*-variation norm of *f*, that is, $(V^{p} (f,[a,b] ) )^{1/p}$, is replaced by the factor $(V^{p} (f,[a,b] ) )^{1-1/q} \Vert f \Vert _{\text{osc},[a,b]}^{1+p/q-p}$, which may be many times smaller than this norm. We formulate an even more improved version of the Loéve-Young inequality in Remark 2. The inequality formulated in Remark 2, together with relation (18), yields (4), and this, together with (5), yields (3), but reasoning in opposite direction (derivation of (4) or inequality stated in Remark 2 from (3)) seems to be not possible.

These results may be applied, for example, when *f* and *g* are trajectories of *α*-stable processes $X^{1}$, $X^{2}$ with $\alpha\in(1,2)$. However, since the obtained results are formulated in terms of rate-independent functionals, like the truncated variation or *p*-variation, they remain valid when $f (t )=F (X^{1} (A (t ) ) )$ and $g (t )=G (X^{2} (B (t ) ) )$ (with the technical assumption that the jumps of *f* and *g* do not occur at the same time), where $A,B: [0,+\infty )\rightarrow [0,+\infty )$ are piecewise monotonic, possibly random, changes of time (*i.e*. there exist $0=T_{0}< T_{1}<\cdots$ such that $T_{n}\rightarrow +\infty $ almost surely as $n\rightarrow +\infty $, and *A* and *B* are monotonic on each interval $(T_{i-1},T_{i} )$, $i=1,2,\ldots$), and $F,G:\mathbb{R}\rightarrow \mathbb{R}$ are locally Lipschitz.

As it was already mentioned, it appears that it is possible to derive weaker conditions under which the improved Loéve-Young inequality still holds, and we will prove that it still holds (and the Riemann-Stieltjes integral $\int _{a}^{b}f\,\mathrm{d}g$ exists) for functions *f* and *g* with no common points of discontinuity, satisfying

$$\sup_{\delta>0}\delta^{p-1} \operatorname{TV}^{\delta} \bigl(f,[a,b]\bigr) < \infty \quad\text{and}\quad\sup_{\delta>0} \delta^{q-1} \operatorname{TV}^{\delta}\bigl(g,[a,b]\bigr) < \infty , $$

respectively. Moreover, in such a case the indefinite integral $I (t ):=\int _{a}^{t}f\,\mathrm{d}g$ reveals similar irregularity as the integrator *g*, namely, $\sup_{\delta>0}\delta^{q-1} \operatorname{TV}^{\delta}(I,[a,b]) <\infty $. We will also prove that, for any $p \ge1$, the class of functions $f:[a,b]\rightarrow W$, where *W* is a Banach space, such that $\operatorname{TV}^{\delta}(f,[a,b]) =O (\delta^{1-p} )$ as $\delta \rightarrow 0+$ is a Banach space. We denote it by $\mathcal {U}^{p}([a,b],W)$. The property $f \in\mathcal{U}^{p}([a,b],W)$ is weaker than the finiteness of *p*-variation but stronger than the finiteness of *q*-variation for all $q>p$.

From early work of Lyons [15] it is well known that whenever *f* and *g* have finite *p*- and *q*-variations, respectively, $p > 1$, $q > 1$ and $p^{-1}+q^{-1}>1$, then the indefinite integral $I(\cdot)$ has finite *q*-variation. However, it is also well known that a symmetric *α*-stable process *X* with $\alpha \in[1,2]$ has finite *p*-variation for any $p>\alpha$ whereas its *α*-variation is infinite (on any proper compact subinterval of $[0,+\infty )$); see, for example, [16, Thm. 4.1]. Thus, if, for example, $f (t )=F (X^{1} (A (t ) ) )$ and $g (t )=G (X^{2} (B (t ) ) )$ are like in the former paragraph, then we can say that $I (\cdot )$ has finite *p*-variation on any compact subinterval of $[0,+\infty )$ for any $p>\alpha$ but cannot say much more. From our results it will follow that $I (\cdot)\in\mathcal {U}^{\alpha}([0,t],\mathbb{R})$ for any ${t\ge0}$; moreover, we will get estimates for *I* stronger than those already known; see Theorem 3. Thus, the introduction of the new spaces and inequalities has at least two applications: (1) we identify the family of functions with finite *p*-variation as a proper subset of $\mathcal{U}^{p}$, that is, the family of functions that can be uniformly approximated with accuracy *δ* by simple functions whose total variation is of order $\delta ^{1-p}$ as $\delta \rightarrow 0+$, (2) we get better estimates for the integrals driven by irregular paths, which may be used to strengthen some results on the existence of solutions of the differential equations driven by irregular paths; see [2, Section 3] and Section 4.3.

Let us comment on the organization of the paper. In the next section we prove very general estimates for $\inf_{g \in B(f,c/2)} \operatorname{TV}(g,[a,b]) $ for regulated $f:[a,b]\rightarrow E$, where *E* is any metric space, in terms of the truncated variation of *f*. Next, in Section 3, we use the obtained estimates to prove a new theorem on the existence of the Riemann-Stieltjes integral driven by irregular paths in Banach spaces. In the proofs we closely follow [2]. In Section 4, we introduce the Banach spaces $\mathcal{U}^{p}([a,b],W)$, $p\ge1$ (Section 4.1) and in Section 4.2 obtain more exact estimates of the rate-independent irregularity of functions from these spaces (in terms of *ϕ*-variation). In the last subsection we deal with the irregularity of the integrals driven by signals from the spaces $\mathcal{U}^{p}([a,b],W)$, $p\ge1$.

## Estimates for the variational problem

Let $(E,d )$ be a metric space with metric *d*. For given reals $a< b$, we say that the function $f:[a,b]\rightarrow E$ is *regulated* if it has right limits $f (t+ )$ for any $t\in[a,b )$ and left limits $f (t- )$ for any $t\in(a,b ]$. If *E* is complete, then a necessary and sufficient condition for *f* to be regulated is that it is a uniform limit of step functions (see [17, Thm. 7.6.1]).

Let $f: [a,b ]\rightarrow E$ be regulated. For $c>0$, let us consider the family $B (f,c/2 )$ of *all* functions $g:[a,b]\rightarrow E$ such that $\sup_{t\in[a,b]}d (f (t ),g (t ) )\le c/2$. We will be interested in the following variational problem: find

6 $$ \inf_{g\in B (f,c/2 )} \operatorname{TV}\bigl(g,[a,b]\bigr) , $$

where

$$\operatorname{TV}\bigl(g,[a,b]\bigr) :=\sup_{n}\sup _{a\le t_{0}< t_{1}< \cdots< t_{n}\le b}\sum_{i=1}^{n}d \bigl(g (t_{i} ),g (t_{i-1} ) \bigr). $$

To state our first main result, let us define the *truncated variation* of *g* with the truncation parameter $c \ge0$:

$$\operatorname{TV}^{c}\bigl(g,[a,b]\bigr) :=\sup_{n}\sup _{a\le t_{0}< t_{1}< \cdots< t_{n}\le b}\sum_{i=1}^{n}\max \bigl\{ d \bigl(g (t_{i} ),g (t_{i-1} ) \bigr)-c,0 \bigr\} . $$

Intuitively, the truncated variation with the truncation parameter *c* takes into account only those changes in the values of *g* whose distance is greater than *c*.

### Theorem 1

*For any regulated* $f: [a,b ]\rightarrow E$, *there exists a step function* $f^{c}:[a,b]\rightarrow E$ *such that* $\sup_{t\in[a,b] }d (f (t ),f^{c} (t ) )\le c/2$ *and for any* $\lambda>1$,

$$ \operatorname{TV}\bigl(f^{c},[a,b]\bigr) \le\lambda\cdot \operatorname{TV}^{(\lambda-1)c/(2\lambda)}\bigl(f,[a,b]\bigr) . $$

*Thus the following estimates hold*:

$$\operatorname{TV}^{c}\bigl(f,[a,b]\bigr) \le\inf_{g\in B (f,c/2 )} \operatorname{TV}\bigl(g,[a,b]\bigr) \le\inf_{\lambda>1}\lambda\cdot \operatorname{TV}^{(\lambda-1)c/(2\lambda)}\bigl(f,[a,b]\bigr) . $$

*In particular*, *taking* $\lambda=2$, *we get the double*-*sided estimate*

$$\operatorname{TV}^{c}\bigl(f,[a,b]\bigr) \le\inf_{g\in B (f,c/2 )} \operatorname{TV}\bigl(g,[a,b]\bigr) \le2 \cdot \operatorname{TV}^{c/4}\bigl(f,[a,b] \bigr) . $$

*Moreover*, *if* *E* *is a normed vector space with norm* $\Vert \cdot \Vert _{E}$, *then there exists* $f^{c,\mathrm{lin}}:[a,b]\rightarrow E$ *such that* $f^{c,\mathrm{lin}}$ *is piecewise linear*, *the jumps of* $f^{c,\mathrm{lin}}$ *occur only at the points where the jumps of* *f* *do*, $\sup_{t\in[a,b]} \Vert f (t )-f^{c,\mathrm{lin}} (t ) \Vert _{E}\le c$ *and* $\operatorname{TV}(f^{c,\mathrm{lin}},[a,b]) = \operatorname{TV}(f^{c},[a,b]) $.

### Proof

The estimate from below

$$\inf_{g\in B (f,c/2 )} \operatorname{TV}\bigl(g,[a,b]\bigr) \ge \operatorname{TV}^{c}\bigl(f,[a,b]\bigr) $$

follows immediately from the triangle inequality: if $\sup_{t\in[a,b] }d (f (t ),g (t ) )\le c/2$, then for any $a\le s< t\le b$,

$$\begin{aligned} \max \bigl\{ d \bigl(g (t ),g (s ) \bigr)-c,0 \bigr\} & \le\max \bigl\{ d \bigl(g (t ),g (s ) \bigr)-d \bigl(g (t ),f (t ) \bigr)-d \bigl(g (s ),f (s ) \bigr),0 \bigr\} \\ & \le d \bigl(f (t ),f (s ) \bigr).\end{aligned} $$

The estimate from above follows from the following greedy algorithm. Let us consider the sequence of times defined in the following way: $\tau_{0}=a$ and, for $n=1,2,\ldots$ ,

$$\tau_{n}= \textstyle\begin{cases} \inf\{ t\in(\tau_{n-1},b ]:d (f (t ),f (\tau_{n-1} ) )>c/2 \} \\ \phantom{+\infty \quad}\text{if } {\tau_{n-1}< b} \text{ and } d (f (\tau _{n-1} ),f (\tau_{n-1}+ ) )< c/2; \\ \inf\{ t\in(\tau_{n-1},b ]:d (f (t ),f (\tau_{n-1}+ ) )>c/2 \} \\ \phantom{+\infty \quad}\text{if } {\tau_{n-1}< b} \text{ and } d (f (\tau_{n-1} ),f (\tau _{n-1}+ ) )\ge c/2; \\ +\infty \quad\text{otherwise.} \end{cases} $$

Note that, since *f* is regulated, $\lim_{n\rightarrow +\infty }\tau_{n}=+\infty $. (We apply the convention that $\inf\emptyset=+\infty $.) Now we define a step function $f^{c}\in B (f,c/2 )$ in the following way. For each $n=1,2,\ldots$ such that $\tau_{n-1}< b$, we put

- if $d (f (\tau_{n-1} ),f (\tau_{n-1}+ ) )< c/2$, then
  $$f^{c} (t ):=f (\tau_{n-1} )\quad\mbox{for }t\in[\tau _{n-1},\tau_{n} )\cap[a,b ]; $$
- if $d (f (\tau_{n-1} ),f (\tau_{n-1}+ ) )\ge c/2$, then $f^{c} (\tau_{n-1} ):=f (\tau_{n-1} )$ and
  $$f^{c} (t ):=f (\tau_{n-1}+ )\quad\mbox{for }t\in(\tau _{n-1},\tau_{n} )\cap[a,b ]. $$

This way the function $f^{c}$ is defined for all $t\in[a,b]$.

It is not difficult to see that the just constructed $f^{c}$ satisfies $\sup_{t\in[a,b]}d (f (t ),f^{c} (t ) )\le c/2$, and for each $n=1,2,\ldots$ such that $\tau_{n}\le b$, we have

- if $d (f (\tau_{n-1} ),f (\tau_{n-1}+ ) )< c/2$ then
  7 $$ d \bigl(f^{c} (\tau_{n-1} ),f^{c} ( \tau_{n-1}+ ) \bigr)=0 $$
  and
  8 $$ d \bigl(f^{c} (\tau_{n-1}+ ),f^{c} ( \tau_{n} ) \bigr)=d \bigl(f (\tau_{n-1} ),f ( \tau_{n} ) \bigr)\ge c/2; $$
- if $d (f (\tau_{n-1} ),f (\tau_{n-1}+ ) )\ge c/2$, then
  9 $$ d \bigl(f^{c} (\tau_{n-1} ),f^{c} ( \tau_{n-1}+ ) \bigr)=d \bigl(f (\tau_{n-1}+ ),f ( \tau_{n-1}+ ) \bigr)\ge c/2 $$
  and
  10 $$ d \bigl(f^{c} (\tau_{n-1}+ ),f^{c} ( \tau_{n} ) \bigr)=d \bigl(f (\tau_{n-1}+ ),f ( \tau_{n} ) \bigr)\ge c/2. $$

Let $N=\max\{ n:\tau_{n-1}< b \} $. From the elementary inequality $x\le\lambda\max\{ x-\frac{\lambda-1}{2\lambda}c,0 \} $ (which holds for for $x\in\{ 0 \} \cup[c/2,+\infty )$ and $\lambda>1$) and from (7)-(10) we have

$$\begin{aligned} \operatorname{TV}\bigl(f^{c},[a,b]\bigr) & =\sum _{n=1}^{N} \bigl\{ d \bigl(f^{c} ( \tau_{n-1} ),f^{c} ( \tau_{n-1}+ ) \bigr)+d \bigl(f^{c} (\tau_{n-1}+ ),f^{c} ( \tau_{n}\wedge b ) \bigr) \bigr\} \\ & \le\lambda\sum_{n=1}^{N}\max \biggl\{ d \bigl(f^{c} (\tau_{n-1} ),f^{c} ( \tau_{n-1}+ ) \bigr)-\frac{\lambda -1}{2\lambda}c,0 \biggr\} \\ & \quad+\lambda\sum_{n=1}^{N}\max \biggl\{ d \bigl(f^{c} (\tau_{n-1} ),f^{c} ( \tau_{n}\wedge b ) \bigr)-\frac{\lambda -1}{2\lambda}c,0 \biggr\} \\ & \le\lambda\sum_{n=1}^{N}\max \biggl\{ d \bigl(f (\tau_{n-1} ),f (\tau_{n-1}+ ) \bigr)- \frac{\lambda-1}{2\lambda}c,0 \biggr\} \\ & \quad+\lambda\sum_{n=1}^{N}\max \biggl\{ d \bigl(f (\tau_{n-1}+ ),f (\tau_{n}\wedge b ) \bigr)- \frac{\lambda -1}{2\lambda}c,0 \biggr\} \\ & \le\lambda \operatorname{TV}^{(\lambda-1)c/(2\lambda)}\bigl(f,[a,b]\bigr) . \end{aligned}$$

Thus, since $f^{c}\in B (f,c/2 )$ and *λ* was an arbitrary number from the interval $(1,+\infty )$, we have

$$\inf_{g\in B (f,c/2 )} \operatorname{TV}\bigl(g,[a,b]\bigr) \le \operatorname{TV} \bigl(f^{c},[a,b]\bigr) \le\inf_{\lambda>1}\lambda\cdot \operatorname{TV}^{(\lambda-1)c/(2\lambda)}\bigl(f,[a,b]\bigr) . $$

The construction of the function $f^{c,\mathrm{lin}}$ is similar. For $\tau_{n}$, $n=0,1,\ldots$ , such that $\tau_{n}\le b$, we define $f^{c,\mathrm{lin}} (\tau_{n} )=f (\tau_{n} )$, and for $t\in(\tau_{n-1},\tau_{n} )\cap[a,b]$, $n=0,1,\ldots$ such that $\tau_{n-1}< b$, we defined it as follows.

- If $d (f (\tau_{n-1} ),f (\tau_{n-1}+ ) )< c/2$, $\tau_{n}\le b$ and $f (\tau_{n}- )\neq f (\tau_{n} )$, then for $t\in(\tau_{n-1},\tau_{n} )$, we put
  $$f^{c,\mathrm{lin}} (t ):=f (\tau_{n-1} ); $$
- if $d (f (\tau_{n-1} ),f (\tau_{n-1}+ ) )< c/2$ and $\tau_{n}=+\infty $, then for $t\in(\tau_{n-1},b ]$, we put
  $$f^{c,\mathrm{lin}} (t ):=f (\tau_{n-1} ); $$
- if $d (f (\tau_{n-1} ),f (\tau_{n-1}+ ) )< c/2$, $\tau_{n}\le b$ and $f (\tau_{n}- )=f (\tau_{n} )$, then for $t\in(\tau_{n-1},\tau_{n} )$, we put
  $$f^{c,\mathrm{lin}} (t ):=\frac{\tau_{n}-t}{\tau_{n}-\tau_{n-1}}f (\tau_{n-1} )+ \frac{t-\tau_{n-1}}{\tau_{n}-\tau_{n-1}}f (\tau_{n} ); $$
- if $d (f (\tau_{n-1} ),f (\tau_{n-1}+ ) )\ge c/2$, $\tau_{n}\le b$ and $f (\tau_{n}- )\neq f (\tau_{n} )$, then for $t\in(\tau_{n-1},\tau_{n} )$, we put
  $$f^{c,\mathrm{lin}} (t ):=f (\tau_{n-1}+ ); $$
- if $d (f (\tau_{n-1} ),f (\tau_{n-1}+ ) )\ge c/2$ and $\tau_{n}=+\infty $, then for $t\in(\tau_{n-1},b ]$ we put
  $$f^{c,\mathrm{lin}} (t ):=f (\tau_{n-1}+ ); $$
- if $d (f (\tau_{n-1} ),f (\tau_{n-1}+ ) )\ge c/2$, $\tau_{n}\le b$ and $f (\tau_{n}- )=f (\tau_{n} )$ then for $t\in(\tau_{n-1},\tau_{n} )$,
  $$f^{c,\mathrm{lin}} (t ):=\frac{\tau_{n}-t}{\tau_{n}-\tau_{n-1}}f (\tau_{n-1}+ )+ \frac{t-\tau_{n-1}}{\tau_{n}-\tau_{n-1}}f (\tau_{n} ). $$

It is straightforward to verify that $\sup_{t\in[a,b]} \Vert f (t )-f^{c,\mathrm{lin}} (t ) \Vert _{E}\le c$, $\operatorname{TV}(f^{c,\mathrm{lin}},[a,b]) = \operatorname{TV}(f^{c},[a,b]) $ and the jumps of $f^{c,\mathrm{lin}}$ occur only at the points where the jumps of *f* do. □

## Integration of irregular signals in Banach spaces

Directly from the definition it follows that the truncated variation is a superadditive functional of the interval, that is, for $c\geq0$ and $d\in(a,b )$,

11 $$ \operatorname{TV}^{\delta}\bigl(f,[a,b]\bigr) \geq \operatorname{TV}^{\delta} \bigl(f,[a;d]\bigr) + \operatorname{TV}^{\delta}\bigl(f,[d,b]\bigr) . $$

Moreover, if $(E, \Vert \cdot \Vert _{E} )$ is a normed vector space (with norm $\Vert \cdot \Vert _{E}$), then we also have the following easy estimate of the truncated variation of a function $g:[a,b]\rightarrow E$ perturbed by some other function $h:[a,b]\rightarrow E$:

12 $$ \operatorname{TV}^{\delta}\bigl(g+h,[a,b]\bigr) \leq \operatorname{TV}^{\delta} \bigl(g,[a,b]\bigr) + \operatorname{TV}^{0}\bigl(h,[a,b]\bigr) , $$

which stems directly from the inequality

$$\begin{aligned} & \max \bigl\{ \bigl\Vert g (t )+h (t )- \bigl\{ g (s )+h (s ) \bigr\} \bigr\Vert _{E}-\delta,0 \bigr\} \\ & \quad\le\max \bigl\{ \bigl\Vert g (t )-g (s ) \bigr\Vert _{E}- \delta,0 \bigr\} + \bigl\Vert h (t )-h (s ) \bigr\Vert _{E},\quad a\leq s< t\leq b. \end{aligned}$$

Let now $(E, \Vert \cdot \Vert _{E} )$, $(W, \Vert \cdot \Vert _{W} )$ be Banach spaces, let $(V, \Vert \cdot \Vert _{V} )$ be another Banach space, and let $(L (E,V ), \Vert \cdot \Vert _{L (E,V )} )$ be the space of continuous linear mappings $F:E\rightarrow V$ with the norm $\Vert F \Vert _{L (E,V )}=\sup_{e\in E: \Vert e \Vert _{E}=1} \Vert F\cdot e \Vert _{V}$. Throughout the rest of this paper, we will assume that $f:[a,b]\rightarrow W$ and $g:[a,b]\rightarrow E$. We will often encounter the situation where $W=L (E,V )$.

Relations (11) and (12), together with Theorem 1, will allow us to establish a new result on the existence of the Riemann-Stieltjes integral with the integrand $f:[a,b]\rightarrow L (E,V )$ and the integrator *g*. By saying that the Riemann-Stieltjes integral exists we will mean that for any sequence of partitions $\pi^{n}= \{ a=t_{0}^{n}< t_{1}^{n}<\cdots<t_{k(n)}^{n}=b \} $ such that $\operatorname {mesh}(\pi^{n}):=\max_{i=1,2,\ldots, k(n)} (t_{i}^{n} - t_{i-1}^{n}) \rightarrow 0$ as $n \rightarrow +\infty $ and for any $\xi_{i}^{n} \in[t_{i-1}^{n}, t_{i}^{n}]$, the Riemann-Stieltjes sums

$$\sum_{i=1}^{k(n)} f \bigl( \xi_{i}^{n} \bigr) \bigl[g\bigl(t_{i}^{n} \bigr) - g\bigl(t_{i-1}^{n}\bigr)\bigr] $$

converge, and the limit denoted by $\int_{a}^{b}f\,\mathrm{d}g$ is independent of the choice of $\xi_{i}^{n}$. (For $\xi,s,t\in[a,b]$, by $f (\xi) [g (t )-g (s ) ]$ we mean the value of the linear mapping $f (\xi)$ evaluated at the vector $g (t )-g (s )$, that is, the element of the space *V*.)

### Theorem 2

*Let* $f:[a,b]\rightarrow L (E,V )$ *and* $g:[a,b]\rightarrow E$ *be two regulated functions that have no common points of discontinuity*. *Let* $\eta_{0}\geq\eta _{1}\geq\cdots$ *and* $\theta_{0}\geq\theta_{1}\geq\cdots$ *be two sequences of positive numbers such that* $\eta_{k}\downarrow0$, $\theta_{k}\downarrow0$ *as* $k\rightarrow+\infty$. *Define* $\eta_{-1}:=\frac{1}{2}\sup_{a\leq t\leq b} \Vert f (t )-f (a ) \Vert _{L (E,V )}$ *and*

$$S:=4\sum_{k=0}^{+\infty}3^{k} \eta_{k-1}\cdot \operatorname{TV}^{\theta_{k}/4}\bigl(g,[a,b]\bigr) +4\sum _{k=0}^{\infty}3^{k} \theta_{k}\cdot \operatorname{TV}^{\eta_{k}/4}\bigl(f,[a,b]\bigr) . $$

*If* $S<+\infty$, *then the Riemann*-*Stieltjes integral* $\int _{a}^{b}f\,\mathrm{d}g$ *exists*, *and we have the following estimate*,

13 $$ \biggl\Vert \int_{a}^{b}f\,\mathrm{d}g-f (a ) \bigl[g (b )-g (a ) \bigr] \biggr\Vert _{V}\leq S. $$

The proof of Theorem 2 is based on the following lemmas.

### Lemma 1

(Summation by parts in a Banach space)

*Let* $f:[a,b]\rightarrow L (E,V )$, $g:[a,b]\rightarrow E$, *and let* $c=t_{0}< t_{1}<\cdots<t_{n}=d$ *be any partition of the interval* $[c,d ]\subset[a,b]$. *Let* $\xi_{0}=c$ *and* $\xi_{1},\ldots,\xi_{n}$ *be such that* $t_{i-1}\leq\xi_{i}\leq t_{i}$ *for* $i=1,2,\ldots,n$. *Then*

14 $$ \sum_{i=1}^{n} \bigl[f ( \xi_{i} )-f (c ) \bigr] \bigl[g (t_{i} )-g (t_{i-1} ) \bigr]=\sum_{i=1}^{n} \bigl[f (\xi_{i} )-f (\xi_{i-1} ) \bigr] \bigl[g (d )-g (t_{i-1} ) \bigr]. $$

### Proof

For $i=1,2,\ldots,n$, let us denote $f_{i}=f (\xi _{i} )-f (\xi_{i-1} )$ and $g_{i}=g (t_{i} )-g (t_{i-1} )$. We have

$$\begin{aligned} \sum_{i=1}^{n} \bigl[f ( \xi_{i} )-f (c ) \bigr] \bigl[g (t_{i} )-g (t_{i-1} ) \bigr] &=\sum_{i=1}^{n} \Biggl(\sum_{j=1}^{i}f_{j} \Biggr)g_{i}=\sum_{j=1}^{n}f_{j} \Biggl( \sum_{i=j}^{n}g_{i} \Biggr) \\ &= \sum_{i=1}^{n} \bigl[f (\xi_{i} )-f (\xi_{i-1} ) \bigr] \bigl[g (d )-g (t_{i-1} ) \bigr]. \end{aligned}$$

 □

### Lemma 2

*Let* $f:[a,b]\rightarrow L (E,V )$ *and* $g:[a,b]\rightarrow E$ *be two regulated functions*. *Let* $c=t_{0}< t_{1}<\cdots<t_{n}=d$ *be any partition of the interval* $[c,d ]\subset[a,b]$, *and let* $\xi_{0}=c$ *and* $\xi_{1},\ldots,\xi_{n}$ *be such that* $t_{i-1}\leq\xi_{i}\leq t_{i}$ *for* $i=1,2,\ldots,n$. *Then for* $\delta_{-1}:=\frac{1}{2}\sup_{c\leq t\leq d} \Vert f (t )-f (c ) \Vert _{L (E,V )}$ *and any* $\delta_{0}\geq\delta_{1}\geq\cdots\geq\delta_{r}>0$ *and* $\varepsilon_{0}\geq\varepsilon_{1}\geq\cdots\geq\varepsilon_{r}>0$, *the following estimate holds*:

$$\begin{aligned} & \Biggl\Vert \sum_{i=1}^{n}f ( \xi_{i} ) \bigl[g (t_{i} )-g (t_{i-1} ) \bigr]-f (c ) \bigl[g (d )-g (c ) \bigr] \Biggr\Vert _{V} \\ & \quad \leq4\sum_{k=0}^{r}3^{k} \delta_{k-1}\cdot \operatorname{TV}^{\varepsilon_{k}/4}\bigl(g,[c,d]\bigr) +4\sum _{k=0}^{r}3^{k} \varepsilon_{k}\cdot \operatorname{TV}^{\delta_{k}/4}\bigl(f,[c,d]\bigr) +n \delta_{r}\varepsilon_{r}. \end{aligned}$$

### Proof

The proof goes exactly along the same lines as the proof of [2, Lemma 1] with the obvious changes. The idea is to utilize Theorem 1 and approximate the functions *g* an *f* by two piecewise linear functions $g^{\varepsilon_{0}}:[a,b]\rightarrow E$ and $f^{\delta_{0}}:[a,b]\rightarrow L (E,V )$ satisfying the following conditions:

15 $$ \sup_{t\in[c,d ]} \bigl\Vert g (t )-g^{\varepsilon _{0}} (t ) \bigr\Vert _{E}\leq\varepsilon_{0}\quad\mbox{and}\quad \operatorname{TV} \bigl(g^{\varepsilon_{0}},[c,d]\bigr) \le2 \operatorname{TV}^{\varepsilon_{0}/4}\bigl(g,[c,d ] \bigr) $$

and

16 $$ \sup_{t\in[c,d ]} \bigl\Vert f (t )-f^{\delta _{0}} (t ) \bigr\Vert _{L (E,V )}\leq\delta_{0}\quad\mbox{and} \quad\operatorname{TV} \bigl(f^{\delta_{0}},[c,d]\bigr) \le2 \operatorname{TV}^{\delta_{0}/4}\bigl(f,[c,d ] \bigr) . $$

Define $g_{0} := g$, $f_{0} := f$, $g_{1} := g_{0} - g_{0}^{\varepsilon_{0}}$, $f_{1} := f_{0} - f_{0}^{\delta_{0}}$, and for $k=2,\ldots,r$, define $g_{k}:=g_{k-1}-g_{k-1}^{\varepsilon_{k-1}}$ and $f_{k}:=f_{k-1}-f_{k-1}^{\delta_{k-1}}$ similarly as $g_{1}$ and $f_{1}$. Repeating the same arguments as in the proof of [2, Lemma 1] (multiple application of the summation by parts), we get

17 $$\begin{aligned}[b] & \Biggl\Vert \sum_{i=1}^{n}f ( \xi_{i} ) \bigl[g (t_{i} )-g (t_{i-1} ) \bigr]-f (c ) \bigl[g (d )-g (c ) \bigr] \Biggr\Vert _{V} \\ & \quad \leq4\sum_{k=0}^{r} \delta_{k-1}\cdot \operatorname{TV}^{\varepsilon_{k}/4}\bigl(g_{k},[c,d] \bigr) +4\sum_{k=0}^{r} \varepsilon_{k} \cdot \operatorname{TV}^{\delta_{k}/4}\bigl(f_{k},[c,d] \bigr) +4n \delta_{r}\varepsilon_{r} \end{aligned} $$

and, for $k=1,2,\ldots,r$,

$$\operatorname{TV}^{\varepsilon_{k}/4}\bigl(g_{k},[c,d]\bigr) \leq3^{k} \operatorname{TV}^{\varepsilon_{k}/4}\bigl(g,[c,d]\bigr) $$

and

$$\operatorname{TV}^{\delta_{k}/4}\bigl(f_{k},[c,d]\bigr) \leq3^{k} \operatorname{TV}^{\delta_{k}/4}\bigl(f,[c,d]\bigr) . $$

By (17) and the last two estimates we get the desired estimate. □

Now we proceed to the proof of Theorem 2.

### Proof of Theorem 2

Again, the proof goes exactly along the same lines as the proof of [2, Thm. 1] with the obvious changes. □

### An improved version of the Loéve-Young inequality

Now we will obtain an improved version of the Loéve-Young inequality for integrals driven by irregular signals attaining their values in Banach spaces. Our main tool will be Theorem 2 and the following simple relation between the rate of growth of the truncated variation and finiteness of *p*-variation. If $V^{p} (f,[a,b])<+\infty$ for some $p\ge1$, then for every $\delta>0$,

18 $$ \operatorname{TV}^{\delta}\bigl(f,[a,b]\bigr) \leq V^{p} \bigl(f,[a,b] \bigr)\delta^{1-p}. $$

This result follows immediately from the elementary estimate: for any $x \ge0$,

$$\delta^{p-1}\max\{ x-\delta,0 \} \le \textstyle\begin{cases} 0 & \mbox{if }x\le\delta\\ x^{p} & \mbox{if }x>\delta \end{cases}\displaystyle \le x^{p}. $$

Notice also that if $V^{p} (f,[a,b])<+\infty$ for some $p>0$, then *f* is regulated. For $p\ge1$ and a Banach space *W*, by $\mathcal {V}^{p}([a,b],W)$ we denote the Banach space of all functions $f:[a,b]\rightarrow W$ such that $V^{p} (f,[a,b])<+\infty$. By $\Vert f \Vert _{p\text{-var},[a,b]}$ we denote the seminorm

$$\Vert f \Vert _{p\text{-var},[a,b]}:= \bigl(V^{p} \bigl(f,[a,b]\bigr) \bigr)^{1/p}. $$

#### Corollary 1

*Let* $f:[a,b]\rightarrow L (E,V )$ *and* $g:[a,b]\rightarrow E$ *be two functions with no common points of discontinuity*. *If* $f\in\mathcal {V}^{p}([a,b],L (E,V ))$ *and* $g\in\mathcal{V}^{q}([a,b],E)$, *where* $p > 1$, $q > 1$, $p^{-1}+q^{-1}>1$, *then the Riemann*-*Stieltjes integral* $\int_{a}^{b}f\,\mathrm{d}g$ *exists*. *Moreover*, *there exists a constant* $C_{p,q}$, *depending on* *p* *and* *q* *only*, *such that*

$$\biggl\Vert \int_{a}^{b}f\,\mathrm{d}g-f (a ) \bigl[g (b )-g (a ) \bigr] \biggr\Vert _{V}\leq C_{p,q} \Vert f \Vert _{p\textit{-}\mathrm{var},[a,b]}^{p-p/q} \Vert f \Vert _{\mathrm {osc},[a,b]}^{1+p/q-p} \Vert g \Vert _{q\textit{-}\mathrm{var},[a,b]}. $$

#### Proof

By Theorem 2 it suffices to prove that, for some positive sequences $\eta_{0}\geq\eta_{1}\geq \cdots$ and $\theta_{0}\geq\theta_{1}\geq\cdots$ such that $\eta _{k}\downarrow0$, $\theta_{k}\downarrow0$ as $k\rightarrow+\infty$, and $\eta _{-1}=\sup_{a\leq t\leq b} \Vert f (t )-f (a ) \Vert _{L (E,V )}$, we have

$$\begin{aligned} S& : =4\sum_{k=0}^{+\infty}3^{k} \eta_{k-1}\cdot \operatorname{TV}^{\theta_{k}/4}\bigl(g,[a,b]\bigr) +4\sum _{k=0}^{+\infty}3^{k} \theta_{k}\cdot \operatorname{TV}^{\eta_{k}/4}\bigl(f,[a,b]\bigr) \\ & \leq C_{p,q} \Vert f \Vert _{p\text{-var},[a,b]}^{p-p/q} \Vert f \Vert _{\text{osc},[a,b]}^{1+p/q-p} \Vert g \Vert _{q\text{-var},[a,b]}. \end{aligned}$$

The proof will follow from the proper choice of the sequences $(\eta_{k} )$ and $(\theta_{k} )$. Choose

$$\begin{gathered} \alpha=\frac{\sqrt{(q-1)(p-1)}+1}{2},\qquad\beta=\frac{1}{2}\sup_{a\leq t\leq b} \bigl\Vert f (t )-f (a ) \bigr\Vert _{L (E,V )}, \\\gamma= \bigl(V^{q} \bigl(g,[a,b] \bigr) /V^{p} \bigl(f,[a,b] \bigr) \bigr)^{1/q}\beta^{p/q} \end{gathered}$$

and for $k=0,1,\ldots$ , define

$$\eta_{k-1}=\beta\cdot3^{- (\alpha^{2}/ [ (q-1 ) (p-1 ) ] )^{k}+1} $$

and

$$\theta_{k}=\gamma\cdot3^{- (\alpha^{2}/ [ (q-1 ) (p-1 ) ] )^{k}\alpha/ (q-1 )}. $$

By (18), similarly as in the proof of [2, Cor. 2], we estimate

$$\begin{aligned} S & \leq C_{p,q} \bigl(V^{q} \bigl(g,[a,b] \bigr) \bigr)^{1/q} \bigl(V^{p} \bigl(f,[a,b] \bigr) \bigr)^{1-1/q}\beta^{1+p/q-p} \\ & \leq C_{p,q} \Vert g \Vert _{q\text{-var},[a,b]} \Vert f \Vert _{p\text{-var},[a,b]}^{p-p/q} \Vert f \Vert _{\text{osc},[a,b]}^{1+p/q-p} \end{aligned}$$

with

$$\begin{aligned} C_{p,q} & = 4^{q} \sum_{k=0}^{+\infty}3^{k+1- (1-\alpha) (\alpha^{2}/ [ (q-1 ) (p-1 ) ] )^{k}} \\ & \quad+ 4^{p}\sum_{k=0}^{+\infty}3^{k+1-p-\alpha(1-\alpha ) (\alpha^{2}/ [ (q-1 ) (p-1 ) ] )^{k}/ (q-1 )} . \end{aligned}$$

 □

## Spaces $\mathcal{U}^{p}([a,b],W)$

### $\mathcal{U}^{p}([a,b],W)$ as a Banach space

Let $p\ge1$, and let *W* be a Banach space. In this subsection, we will prove that the family $\mathcal{U}^{p}([a,b],W)$ of functions $f:[a,b]\rightarrow W$ such that $\sup_{\delta>0}\delta^{p-1} \operatorname{TV}^{\delta}(f,[a,b]) <+\infty $ is a Banach space and that the functional

$$\Vert \cdot \Vert _{p\text{-TV},[a,b]}:\mathcal{U}^{p}\bigl([a,b]\bigr) \rightarrow [0,+\infty ) $$

defined by

19 $$ \Vert f \Vert _{p\text{-TV},[a,b]}:= \Bigl(\sup_{\delta >0} \delta^{p-1} \operatorname{TV}^{\delta}\bigl(f,[a,b]\bigr) \Bigr)^{1/p} $$

is a seminorm on this space (whereas the functional $\Vert f \Vert _{\operatorname{TV},p,[a,b]}= \Vert f (a ) \Vert _{W}+ \Vert f \Vert _{p\text{-TV},[a,b]}$ is a norm). From (18) it follows that

20 $$ \Vert f \Vert _{p\text{-TV},[a,b]} \le \Vert f \Vert _{p\text{-var},[a,b]}, $$

and thus $\mathcal{V}^{p}([a,b],W)\subset\mathcal{U}^{p}([a,b],W)$. It appears that this inclusion is strict. For example, if $0\le a< b$, then a real symmetric *α*-stable process *X* with $\alpha\in(1,2]$ has finite *p*-variation for $p>\alpha$, whereas (as it was already mentioned in the Introduction) its *α*-variation is a.s. infinite (on any proper compact subinterval of $[0,+\infty )$). On the other hand, trajectories of *X* belong a.s. to $\mathcal{U}^{\alpha }([0,t],\mathbb{R})$ for any $t\ge0$; see [18]. For another example, see [19, Thm. 17].

From the results of the next subsection it will also follow that

$$\mathcal{U}^{p}\bigl([a,b],W\bigr)\subset\bigcap _{q>p}\mathcal{V}^{q}\bigl([a,b],W\bigr), $$

but, again, this inclusion is strict.

#### Remark 2

For further justifcation of the importance of the spaces $\mathcal{U}^{p}([a,b],W)$, $p > 1$, let us also notice that if $W=L (E,V )$, *f* belongs to $\mathcal{U}^{q}([a,b],W)$, *g* belongs to $\mathcal{U}^{q}([a,b],E)$ for some $q > 1$ such that $p^{-1}+q^{-1}>1$, and *f* and *g* have no common points of discontinuity, then the integral $\int_{a}^{b}f\,\mathrm{d}g$ still exists, and we have the estimate

$$\biggl\Vert \int_{a}^{b}f\,\mathrm{d}g-f (a ) \bigl[g (b )-g (a ) \bigr] \biggr\Vert _{V}\leq C_{p,q} \Vert f \Vert _{p-TV,[a,b]}^{p-p/q} \Vert f \Vert _{\mathrm {osc},[a,b]}^{1+p/q-p} \Vert g \Vert _{q\text{-TV},[a,b]} $$

with the same constant $C_{p,q}$ that appears in Corollary 1. This follows from the fact that in the proof of Corollary 1 we were using only estimate (18), which now may be replaced by the estimate

21 $$ \operatorname{TV}^{\delta}\bigl(f,[a,b]\bigr) \le \Vert f \Vert _{p\text{-TV},[a,b]}^{p} \delta^{1-p} $$

for any $\delta>0$, stemming directly from the definition of the norm $\Vert \cdot \Vert _{p\text{-TV},[a,b]}$.

#### Proposition 1

*For any* $p\geq1$, *the functional* $\Vert \cdot \Vert _{p\textit{-}\mathrm{TV},[a,b]}$ *is a seminorm*, *and the functional* $\Vert \cdot \Vert _{\mathrm {TV},p,[a,b]}$ *is a norm on* $\mathcal{U}^{p}([a,b],W)$. $\mathcal{U}^{p}([a,b],W)$ *equipped with this norm is a Banach space*.

#### Proof

For $p=1$, $\Vert \cdot \Vert _{p\text{-TV},[a,b]}$ coincides with $V^{1}(f,[a,b])$, $\Vert \cdot \Vert _{\operatorname{TV},p,[a,b]}$ coincides with the 1-variation norm $\Vert f \Vert _{\text{var},1,[a,b]}:= \Vert f(a) \Vert _{W}+V^{1}(f,[a,b])$, and $\mathcal{U}^{1}([a,b],W)$ is simply the same as the space of functions with bounded total variation. Therefore, for the rest of the proof, we will assume that $p>1$.

The homogeneity of $\Vert \cdot \Vert _{p\text{-TV},[a,b]}$ and $\Vert \cdot \Vert _{\operatorname{TV},p,[a,b]}$ follows easily from the fact that, for $\alpha,\delta>0$, $\operatorname{TV}^{\alpha\delta}(\alpha f,[a,b]) =\alpha \operatorname{TV}^{\delta}(f,[a,b]) $, which is a consequence of the equality

$$\max \bigl\{ \bigl\Vert \alpha f (t )-\alpha f (s ) \bigr\Vert _{W}-\alpha\delta,0 \bigr\} =\alpha\max \bigl\{ \bigl\Vert f (t )-f (s ) \bigr\Vert _{W}-\delta,0 \bigr\} . $$

To prove the triangle inequality, let us take $f,h\in\mathcal{U}^{p}([a,b])$ and fix $\varepsilon>0$. Let $\delta_{0}>0$ and $a\leq t_{0}< t_{1}<\cdots <t_{n}\leq b$ be such that

22 $$ \Biggl(\delta_{0}^{p-1}\sum_{i=1}^{n} \bigl( \bigl\Vert f (t_{i} )-f (t_{i-1} )+h (t_{i} )-h (t_{i-1} ) \bigr\Vert _{W}- \delta_{0} \bigr)_{+} \Biggr)^{1/p} \geq \Vert f+h \Vert _{p\text{-TV};[a,b]}-\varepsilon, $$

where $(\cdot)_{+}:=\max\{ \cdot,0 \} $. By standard calculus, for $x>0$ and $p\geq1$, we have

23 $$ \sup_{\delta>0}\delta^{p-1} (x-\delta)_{+}= \sup_{\delta\geq 0}\delta^{p-1} (x-\delta)=c_{p}x^{p}, $$

where $c_{p}=(p-1)^{p-1}/p^{p}\in[2^{-p};1]$. Denote $x_{0}^{*}=0$ and $x_{i}= \Vert f (t_{i} )-f (t_{i-1} )+h (t_{i} )-h (t_{i-1} ) \Vert _{W}$ for $i=1,2,\ldots,n$. Let $x_{1}^{*}\leq x_{2}^{*}\leq\cdots\leq x_{n}^{*}$ be the nondecreasing rearrangement of the sequence $(x_{i} )$. Notice that by (23) for $\delta\in[x_{j-1}^{*};x_{j}^{*} ]$, $j=1,2,\ldots,n$, we have

$$\begin{aligned} & \delta^{p-1}\sum_{i=1}^{n} \bigl( \bigl\Vert f (t_{i} )-f (t_{i-1} )+h (t_{i} )-h (t_{i-1} ) \bigr\Vert _{W}-\delta \bigr)_{+} \\ & \quad=\delta^{p-1}\sum_{i=j}^{n} \bigl(x_{i}^{*}-\delta \bigr)=\delta^{p-1} \Biggl( \sum_{i=j}^{n}x_{i}^{*}- (n-j+1 )\delta \Biggr) \\ &\quad = (n-j+1 )\delta^{p-1} \biggl(\frac{\sum_{i=j}^{n}x_{i}^{*}}{n-j+1}-\delta \biggr)\leq (n-j+1 )c_{p} \biggl(\frac{\sum_{i=j}^{n}x_{i}^{*}}{n-j+1} \biggr)^{p}. \end{aligned}$$

Hence

24 $$\begin{aligned}[b] & \sup_{\delta>0}\delta^{p-1}\sum _{i=1}^{n} \bigl( \bigl\Vert f (t_{i} )-f (t_{i-1} )+h (t_{i} )-h (t_{i-1} ) \bigr\Vert _{W}-\delta \bigr)_{+} \\ & \quad\leq\max_{j=1,2,\dots,n} (n-j+1 )c_{p} \biggl( \frac{\sum_{i=j}^{n}x_{i}^{*}}{n-j+1} \biggr)^{p}. \end{aligned}$$

On the other hand,

25 $$\begin{aligned}[b] & \sup_{\delta>0}\delta^{p-1}\sum _{i=1}^{n} \bigl( \bigl\Vert f (t_{i} )-f (t_{i-1} )+h (t_{i} )-h (t_{i-1} ) \bigr\Vert _{W}-\delta \bigr)_{+} \\ & \quad=\sup_{\delta>0}\delta^{p-1}\sum _{i=1}^{n} \bigl(x_{i}^{*}- \delta \bigr)_{+}\geq\sup_{\delta>0}\max _{j=1,2,\dots,n}\delta^{p-1}\sum_{i=j}^{n} \bigl(x_{i}^{*}-\delta \bigr) \\ &\quad =\max_{j=1,2,\dots,n}\sup_{\delta>0} \delta^{p-1}\sum_{i=j}^{n} \bigl(x_{i}^{*}-\delta \bigr) \\ &\quad =\max_{j=1,2,\dots,n} (n-j+1 )c_{p} \biggl( \frac{\sum_{i=j}^{n}x_{i}^{*}}{n-j+1} \biggr)^{p}. \end{aligned} $$

By (24) and (25) we get

26 $$\begin{aligned}[b] & \Biggl(\sup_{\delta>0}\delta^{p-1}\sum _{i=1}^{n} \bigl( \bigl\Vert f (t_{i} )-f (t_{i-1} )+h (t_{i} )-h (t_{i-1} ) \bigr\Vert _{W}-\delta \bigr)_{+} \Biggr)^{1/p} \\ &\quad =\max_{j=1,2,\dots,n} (n-j+1 )^{1/p-1}c_{p}^{1/p} \sum_{i=j}^{n}x_{i}^{*}. \end{aligned} $$

Similarly, denoting by $y_{i}^{*}$ and $z_{i}^{*}$ the nondecreasing rearrangements of the sequences $y_{i}= \Vert f (t_{i} )-f (t_{i-1} ) \Vert _{W}$ and $z_{i}= \Vert h (t_{i} )-h (t_{i-1} ) \Vert _{W}$, respectively, we get

$$\begin{aligned} \Vert f \Vert _{p\text{-TV},[a,b]} & \ge \Biggl(\sup_{\delta >0} \delta^{p-1}\sum_{i=1}^{n} \bigl( \bigl\Vert f (t_{i} )-f (t_{i-1} ) \bigr\Vert _{W}-\delta \bigr)_{+} \Biggr)^{1/p} \\ & =\max_{j=1,2,\dots,n} (n-j+1 )^{1/p-1}c_{p}^{1/p} \sum_{i=j}^{n}y_{i}^{*} \end{aligned}$$

and

$$\begin{aligned} \Vert h \Vert _{p\text{-TV},[a,b]} & \ge \Biggl(\sup_{\delta >0} \delta^{p-1}\sum_{i=1}^{n} \bigl( \bigl\Vert h (t_{i} )-h (t_{i-1} ) \bigr\Vert _{W}-\delta \bigr)_{+} \Biggr)^{1/p} \\ & =\max_{j=1,2,\dots,n} (n-j+1 )^{1/p-1}c_{p}^{1/p} \sum_{i=j}^{n}z_{i}^{*}. \end{aligned}$$

By the triangle inequality and the definition of $y_{i}^{*}$ and $z_{i}^{*}$ for $j=1,2,\ldots,n$, we have $\sum_{i=j}^{n}x_{i}^{*}\leq \sum_{i=j}^{n}y_{i}^{*}+\sum_{i=j}^{n}z_{i}^{*}$. Hence

$$\begin{aligned} & \max_{j=1,2,\dots,n} (n-j+1 )^{1/p-1}c_{p}^{1/p} \sum_{i=j}^{n}x_{i}^{*}\\ &\quad\leq\max_{j=1,2,\dots,n} (n-j+1 )^{1/p-1}c_{p}^{1/p} \sum_{i=j}^{n} \bigl(y_{i}^{*}+z_{i}^{*} \bigr) \\ & \quad\leq\max_{j=1,2,\dots,n} (n-j+1 )^{1/p-1}c_{p}^{1/p} \sum_{i=j}^{n}y_{i}^{*}+ \max_{j=1,2,\dots,n} (n-j+1 )^{1/p-1}c_{p}^{1/p} \sum_{i=j}^{n}z_{i}^{*} \\ & \quad\leq \Vert f \Vert _{p\text{-TV},[a,b]}+ \Vert h \Vert _{p\text{-TV},[a,b]}. \end{aligned}$$

Finally, by (22), (26), and the last estimate we get

$$\Vert f+g \Vert _{p\text{-TV},[a,b]}-\varepsilon\leq \Vert f \Vert _{p\text{-TV},[a,b]}+ \Vert g \Vert _{p\text{-TV},[a,b]}. $$

Sending *ε* to 0, we get the triangle inequality for $\Vert \cdot \Vert _{p\text{-TV},[a,b]}$. From this also follows the triangle inequality for $\Vert \cdot \Vert _{\operatorname{TV},p,[a,b]}$.

Now we will prove that the space $\mathcal{U}^{p}([a,b],W)$ equipped with the norm $\Vert \cdot \Vert _{\operatorname{TV},p,[a,b]}$ is a Banach space. To prove this, we need the inequality

27 $$ \operatorname{TV}^{\delta_{1}+\delta_{2}}\bigl(f+g,[a,b]\bigr) \leq \operatorname{TV}^{\delta_{1}} \bigl(f,[a,b]\bigr) + \operatorname{TV}^{\delta_{2}}\bigl(g,[a,b]\bigr) $$

for $\delta_{1},\delta_{2}\geq0$. It follows from the elementary estimate

28 $$ \bigl( \Vert w_{1}-w_{2} \Vert _{W}- \delta_{1}-\delta_{2}\bigr)_{+}\leq\bigl( \Vert w_{1} \Vert _{W}-\delta_{1} \bigr)_{+}+ \bigl( \Vert w_{2} \Vert _{W}- \delta_{2}\bigr)_{+} $$

for $w_{1},w_{2}\in W$ and nonnegative $\delta_{1}$ and $\delta_{2}$. We also have $\operatorname{TV}^{\delta}(f,[a,b]) \geq( \Vert f \Vert _{\text{osc},[a,b]}-\delta)_{+}$. From this and from (23) it follows that

29 $$ \Vert f \Vert _{\operatorname{TV},p,[a,b]}\geq \big\vert f(a) \big\vert +c_{p}^{1/p} \Vert f \Vert _{\text{osc},[a,b]}\geq c_{p}^{1/p} \Vert f \Vert _{\infty ,[a,b]}, $$

where $\Vert f \Vert _{\infty ,[a,b]}:=\sup_{t\in[a,b]} \Vert f (t ) \Vert _{W}$. Hence any Cauchy sequence $(f_{n})_{n=1}^{\infty }$ in $\mathcal {U}^{p}([a,b],W)$ converges uniformly to some $f_{\infty }:[a,b]\rightarrow W$. Assume that $\Vert f_{\infty }-f_{n} \Vert _{\operatorname{TV},p,[a,b]}\nrightarrow0$ as $n\rightarrow +\infty $. Thus, there exist a positive number *κ*, a sequence of positive integers $n_{k}\rightarrow +\infty $ and a sequence of positive reals $\delta_{k}$, $k=1,2,\ldots$ , such that $\delta_{k}^{p-1} \operatorname{TV}^{\delta_{k}}(f_{n_{k}}-f_{\infty },[a,b]) \geq\kappa^{p}$. Let *N* be a positive integer such that

30 $$ \Vert f_{m}-f_{n} \Vert _{\operatorname{TV},p,[a,b]}< \kappa /2^{1-1/p}\quad\text{for }m,n\geq N, $$

and let $k_{0}$ be the minimal positive integer such that $n_{k_{0}}\geq N$. For sufficiently large $n\geq N$, we have $\Vert f_{n}-f_{\infty } \Vert _{\infty ,[a,b]}\leq\delta_{k_{0}}/4$, and hence $\Vert f_{n}-f_{\infty } \Vert _{\text{osc},[a,b]}\leq \delta_{k_{0}}/2$ and

31 $$ \operatorname{TV}^{\delta_{k_{0}}/2}\bigl(f_{n}-f_{\infty },[a,b]\bigr) =0. $$

Now, by (27)

$$\operatorname{TV}^{\delta_{k_{0}}}\bigl(f_{n_{k_{0}}}-f_{\infty },[a,b]\bigr) \leq \operatorname{TV}^{\delta_{k_{0}}/2}\bigl(f_{n_{k_{0}}}-f_{n},[a,b]\bigr) + \operatorname{TV}^{\delta_{k_{0}}/2}\bigl(f_{n}-f_{\infty },[a,b]\bigr) . $$

From this and from (31) we get

$$(\delta_{k_{0}}/2)^{p-1} \operatorname{TV}^{\delta_{k_{0}}/2} \bigl(f_{n_{k_{0}}}-f_{n},[a,b]\bigr) \geq\delta_{k_{0}}^{p-1} \operatorname{TV}^{\delta_{k_{0}}}\bigl(f_{n_{k_{0}}}-f_{\infty },[a,b]\bigr) /2^{p-1} \geq\kappa^{p}/2^{p-1}, $$

but this (recall (19)) contradicts (30). Thus the sequence $(f_{n})_{n=1}^{\infty }$ converges in the $\mathcal {U}^{p}([a,b],W)$ norm to $f_{\infty }$. Since the sequence $(f_{n})_{n=1}^{\infty }$ was chosen in an arbitrary way, this proves that $\mathcal{U}^{p}([a,b],W)$ is complete. □

#### Remark 3

It is easy to see that the space $\mathcal{U}^{p}([a,b],W)$ equipped with the norm $\Vert \cdot \Vert _{\operatorname{TV},p,[a,b]}$ is not separable. To see this, it suffices for two distinct vectors $w_{1}$ and $w_{2}$ from *W* to consider the family of functions $f_{t}:[a,b]\rightarrow \{w_{1},w_{2}\}$, $f_{t}(s):=\textbf{1}_{\{t\}}(s)w_{1}+(1-\textbf{1}_{\{t\}}(s))w_{2}$, $t\in[a,b]$ ($\textbf{1}_{A}$ denotes the indicator function of a set *A*) and apply (29). However, we do not know if the subspace of continuous functions in $\mathcal {U}^{p}([a,b],\mathbb{R})$ is separable.

#### Remark 4

From the triangle inequality for $\Vert \cdot \Vert _{p\text{-TV},[a,b]}$ it follows that it is an subadditive functional of the interval, that is, for any $p\geq1$, $f:[a,b]\rightarrow W$ and $d\in(a,b)$,

$$\Vert f \Vert _{p\text{-TV},[a,b]}\leq \Vert f \Vert _{p\text{-TV}, [a,d ]}+ \Vert f \Vert _{p\text{-TV}, [d,b ]}. $$

To see this, it suffices to consider the decomposition $f(t)=f_{1}(t)+f_{2}(t)$, $f_{1}(t)=\textbf{1}_{[a,d]}(t)f(t)+\textbf{1}_{(d,b]}(t)f(d)$, $f_{2}(t)=\textbf{1}_{(d,b]}(t)f(t)-\textbf{1}_{(d,b]}(t)f(d)$. We naturally have

$$\begin{aligned} \Vert f \Vert _{p\text{-TV},[a,b]}&= \Vert f_{1}+f_{2} \Vert _{p\text{-TV},[a,b]} \\ & \leq \Vert f_{1} \Vert _{p\text{-TV},[a,b]}+ \Vert f_{2} \Vert _{p\text{-TV},[a,b]} \\ & = \Vert f \Vert _{p\text{-TV}, [a,d ]}+ \Vert f \Vert _{p\text{-TV}, [d,b ]}. \end{aligned}$$

However, superadditivity, as a function of the interval, holding for $\Vert \cdot \Vert _{p\text{-var},[a,b]}^{p}=V^{p}(\cdot,[a,b])$ is no more valid for $\Vert \cdot \Vert _{p\text{-TV},[a,b]}^{p}$. To see this, it suffices to consider the function $f:[-1;1]\rightarrow \{ -1,0,1\}$, $f(t)=\textbf{1}_{(-1,1)}(t)-\textbf{1}_{\{1\}}(t)$. We have $\operatorname{TV}^{\delta}(f,[-1,0]) =(1-\delta)_{+}$, $\operatorname{TV}^{\delta}(f,[0,1]) =(2-\delta)_{+}$ and $\operatorname{TV}^{\delta}(f,[-1,1]) =(1-\delta)_{+}+(2-\delta)_{+}$, and hence $\Vert f \Vert _{2\text{-TV}, [-1;1 ]}^{2}=9/8< \Vert f \Vert _{2\text{-TV}, [-1;0 ]}^{2}+ \Vert f \Vert _{2\text{-TV}, [0;1 ]}^{2}=1/4+1$.

### *ϕ*-variation of the functions from the space $\mathcal {U}^{p} ([a,b], W )$

For a (nondecreasing) function $\phi: [0,+\infty )\rightarrow [0,+\infty )$, let us define the *ϕ*-variation of $f:[a,b]\rightarrow W$ as

$$V^{\phi} \bigl(f,[a,b]\bigr):=\sup_{n}\sup _{a\le t_{0}< t_{1}< \cdots < t_{n}\le b} \sum_{i=1}^{n} \phi \bigl( \bigl\Vert f (t_{i} )-f (t_{i-1} ) \bigr\Vert _{W} \bigr). $$

In this subsection, we prove the following result.

#### Proposition 2

*Let* $p\ge1$ *and suppose that* $\phi: [0,+\infty )\rightarrow [0,+\infty )$ *is such that* $\phi(0 )=0$ *and for each* $t>0$, $\phi (t )>0$,

32 $$ \sup_{0< u\leq s\leq2u\leq2t}\frac{\phi(s )}{\phi(u )}< +\infty \quad\textit{and}\quad\sum _{j=0}^{+\infty }2^{pj}\phi \bigl(2^{-j} \bigr)< +\infty . $$

*Then for any function* $f\in\mathcal{U}^{p} ([a,b], W )$, *we have* $\mathrm{V}^{\phi}(f,[a,b]) <+\infty $.

#### Remark 5

The function *ϕ* satisfies the same assumptions as in [20, Prop. 1].

#### Proof

Let *L* be the least positive integer such that $\sup_{t\in[a,b ]} \Vert f (t ) \Vert _{W}\leq2^{L}$. Consider the partition $\pi= \{ a\leq t_{0}< t_{1}<\cdots<t_{n}\leq b \} $ such that $f (t_{i} )\neq f (t_{i-1} )$ for $i=1,2,\ldots,n$ and for $j=0,1,\ldots$ , define

$$I_{j}= \bigl\{ i\in\{ 1,2,\ldots,n \} : \bigl\Vert f (t_{i} )-f (t_{i-1} ) \bigr\Vert _{W}\in \bigl(2^{L-j},2^{L-j+1} \bigr] \bigr\} $$

and $\delta(j ):=2^{L-j-1}$. Naturally, for $i\in I_{j}$,

$$\bigl\Vert f (t_{i} )-f (t_{i-1} ) \bigr\Vert _{W}-\delta(j )\geq\frac{1}{2} \bigl\Vert f (t_{i} )-f (t_{i-1} ) \bigr\Vert _{W}, $$

and since $\{ 1,2,\ldots,n \} =\bigcup_{j=0}^{+\infty}I_{j}$, we estimate

33 $$\begin{aligned} & \sum_{i=1}^{n}\phi \bigl( \bigl\Vert f (t_{i} )-f (t_{i-1} ) \bigr\Vert _{W} \bigr) \\ &\quad= \sum_{j=0}^{+\infty }\sum _{i\in I_{j}}\phi \bigl( \bigl\Vert f (t_{i} )-f (t_{i-1} ) \bigr\Vert _{W} \bigr) \\ &\quad \leq\sum_{j=0}^{+\infty}\sup _{s\in[2^{L-j},2^{L-j+1} ]}\frac{\phi(s )}{2^{L-j}}\sum_{i\in I_{j}} \bigl\Vert f (t_{i} )-f (t_{i-1} ) \bigr\Vert _{W} \\ & \quad\leq\sum_{j=0}^{+\infty}\sup _{s\in[2^{L-j},2^{L-j+1} ]}\frac{\phi(s )}{2^{L-j}}\cdot2\sum _{i\in I_{j}}\max \bigl\{ \bigl\Vert f (t_{i} )-f (t_{i-1} ) \bigr\Vert _{W}-\delta(j ),0 \bigr\} \\ & \quad\leq\sum_{j=0}^{+\infty}\sup _{s\in[2^{L-j},2^{L-j+1} ]}\frac{\phi(s )}{2^{L-j}}\cdot2 \operatorname{TV}^{\delta(j)} \bigl(f,[a,b]\bigr) \end{aligned}$$

34 $$\begin{aligned} & \quad=\sum_{j=0}^{+\infty}\sup _{s\in[2^{L-j},2^{L-j+1} ]}\frac {\phi(s )}{2^{L-j}}\cdot2\cdot\frac{1}{\delta(j )^{p-1}}\delta(j )^{p-1} \operatorname{TV}^{\delta(j )}\bigl(f,[a,b]\bigr) \\ &\quad \leq\sum_{j=0}^{+\infty}\sup _{s\in[2^{L-j},2^{L-j+1} ]}\frac{\phi(s )}{2^{L-j}}\cdot2\cdot\frac{1}{\delta (j )^{p-1}}\sup _{\delta>0} \bigl\{ {\delta}^{p-1}\cdot \operatorname{TV}^{\delta}\bigl(f,[a,b]\bigr) \bigr\} \end{aligned}$$

35 $$\begin{aligned} &\quad =2^{p} \Vert f \Vert _{p\text{-}\operatorname{TV},[a,b]}^{p}\sum _{j=0}^{+\infty }2^{p (j-L )}\sup _{s\in[2^{L-j},2^{L-j+1} ]}\phi(s ). \end{aligned}$$

By the first assumption in (32) we have that, for all $j=0,1,\ldots$ ,

$$\sup_{s\in[2^{L-j},2^{L-j+1} ]}\phi(s )\leq C (\phi,L )\cdot\phi \bigl(2^{L-j} \bigr) $$

for some constant $C (\phi,L )$ depending on *ϕ* and *L* only. Thus, by the second assumption in (32), $\sum_{j=0}^{+\infty }2^{pj}\phi(2^{-j} )<+\infty $, we get

36 $$\begin{aligned}[b] & \sum_{j=0}^{+\infty}2^{p (j-L )}\sup _{s\in [2^{L-j},2^{L-j+1} ]}\phi(s ) \\ &\quad \leq C (\phi,L ) \Biggl\{ \sum_{j=0}^{L-1}2^{p (j-L )} \phi \bigl(2^{L-j} \bigr)+\sum_{j=L}^{+\infty}2^{p (j-L )} \phi \bigl(2^{L-j} \bigr) \Biggr\} \\ & \quad=C (\phi,L ) \Biggl\{ \sum_{j=0}^{L-1}2^{p (j-L )} \phi \bigl(2^{L-j} \bigr)+\sum_{j=0}^{+\infty }2^{pj} \phi \bigl(2^{-j} \bigr) \Biggr\} < +\infty . \end{aligned} $$

Since estimates (35) and (36) do not depend on the partition *π*, taking the supremum over all partitions of the interval $[a,b]$, we get $\mathrm{V}^{\phi}(f,[a,b]) <+\infty $. □

#### Remark 6

From Proposition 2 it immediately follows that $\mathcal {U}^{p} ([a,b],W )\subset\mathcal{V}^{q} ([a,b],W )$ for any $q>p$, since for any $q>p$, $\phi_{q} (x )=x^{q}$ satisfies (32). It is easy to derive more exact results. For example, by standard calculus assumptions (32) hold for

$$\phi_{p,\gamma,1} (x ):=\frac{x^{p}}{(\ln(1+1/x ))^{\gamma}} \quad\text{or}\quad \phi_{p,\gamma,2} (x ):=\frac {x^{p}}{\ln(1+1/x ) (\ln\ln(e+1/x ) )^{\gamma}} $$

when $\gamma>1$. From this we have that $\bigcap_{q>p}\mathcal {V}^{q} ([a,b],W )\neq\mathcal{U}^{q} ([a,b],W )$ since there exist functions $f:[a,b]\rightarrow W$ such that $\mathrm{V}^{\phi_{p,2,1}}(f,[a,b]) =+\infty $ but $f\in\mathcal{V}^{q} ([a,b], W )$ for any $q>p$. An example of such a function is the following. Let ${ w} \in W$ be such that $\Vert { w} \Vert _{W} = 1$. Then $f:[0,1] \rightarrow W$ is defined as

$$f(t)= \textstyle\begin{cases}({\ln n}/{n})^{1/p} { w} & \text{if } t = {1}/{n} \text{ for } n=1,2,\ldots,\\ {0 } & \text{otherwise.} \end{cases} $$

It remains an open question if it is possible to obtain the finiteness of the *ϕ*-variation of functions from $\mathcal{U}^{p} ([a,b], W )$ for *ϕ* vanishing slower (as $x \rightarrow 0+$) than ${x^{p}}/{{\ln(1+1/x )}} $.

### Irregularity of the integrals driven by functions from $\mathcal{U}^{p} ([a,b],W )$

In [15, Section 2], there are considered $\Vert \cdot \Vert _{p\text{-var}, [a,b ]}$ norms of the integrals of the form $[a,b]\ni t\mapsto\int _{a}^{t}f\,\mathrm{d}g$, with $f\in\mathcal{V}^{p}([a,b],L (E,V ))$ and $g\in \mathcal{V}^{q}([a,b],E)$, where $p>1$, $q>1$ and $p^{-1}+q^{-1}>1$. Now, we turn to investigate the $\Vert \cdot \Vert _{p\text{-TV}, [a,b ]}$ norms of similar integrals, but for $f\in\mathcal{U}^{p}([a,b],L (E,V ))$ and $g\in\mathcal{U}^{q}([a,b],W)$. This, in view of the preceding subsection, will give us more exact results about the irregularity of the indefinite integrals $\int _{a}^{\cdot}f\,\mathrm{d}g$. We will prove the following:

#### Theorem 3

*Assume that* $f\in\mathcal{U}^{p}([a,b],L (E,V ))$ *and* $g\in \mathcal{U}^{q}([a,b],E)$ *for some* $p>1$ *and* $q>1$ *such that* $p^{-1}+q^{-1}>1$ *and they have no common points of discontinuity*. *Then there exists a constant* $D_{p,q}<+\infty$, *depending on* *p* *and* *q* *only*, *such that*

$$\biggl\Vert \int_{a}^{\cdot} \bigl[f (s )-f (a ) \bigr]\,\mathrm{d}g (s ) \biggr\Vert _{q\textit{-}\mathrm{TV}, [a,b ]}\leq D_{p,q} \Vert f \Vert _{p\textit{-}\mathrm{TV}, [a,b ]}^{p-p/q} \Vert f \Vert _{\mathrm{osc}, [a,b ]}^{1+p/q-p} \Vert g \Vert _{q\textit{-}\mathrm{TV}, [a,b ]}. $$

One more application of Theorem 3 is the following. Assume that $y: [a, b ] \rightarrow E$ is a solution of the equation of the form

37 $$ y(t) = x_{0} + \int_{a}^{t} F\bigl(s,y(s)\bigr)\, \mathrm{d}x(s), $$

where $x\in\mathcal{U}^{q}([a,b],E)$ is continuous, and $F(\cdot,y(\cdot)) \in\mathcal {U}^{p}([a,b],L(E,V))$ for some $p>1$ and $q>1$ such that $p^{-1}+q^{-1} >1$; from this by Theorem 3 we will obtain that $y \in \mathcal{U}^{q}([a,b],E)$.

In our case we have no longer the supperadditivity property of the functional $\Vert \cdot \Vert _{p\text{-TV}, [a,b ]}^{p}$ as the function of interval (see Remark 4), and hence the method of the proof of Theorem 3 will be different from those of related estimates in [15]. It will be similar to the proof of Corollary 1. We need the following lemma.

#### Lemma 3

*Let* $f:[a,b]\rightarrow L (E,V )$ *and* $g:[a,b]\rightarrow E$ *be two regulated functions that have no common points of discontinuity*, *and let* $\delta_{0}\geq\delta_{1}\geq \cdots$, $\varepsilon_{0}\geq\varepsilon_{1}\geq\cdots$ *be two sequences of nonnegative numbers such that* $\delta_{k}\downarrow0$, $\varepsilon _{k}\downarrow0$ *as* $k\rightarrow+\infty$. *Assume that*, *for* $\delta_{-1}:=\frac {1}{2}\sup_{a\leq t\leq b} \Vert f (t )-f (a ) \Vert _{L(E,V)}$ *and*

$$S=4\sum_{k=0}^{+\infty}3^{k} \delta_{k-1}\cdot \operatorname{TV}^{\varepsilon_{k}}\bigl(g,[a,b ]\bigr) +4\sum _{k=0}^{\infty}3^{k} \varepsilon_{k}\cdot \operatorname{TV}^{\delta_{k}}\bigl(f,[a,b]\bigr) , $$

*we have* $S<+\infty $. *Defining*

$$\gamma:=8\sum_{k=0}^{+\infty}3^{k} \varepsilon_{k}\cdot \operatorname{TV}^{\delta_{k}}\bigl(f,[a,b]\bigr) , $$

*we get*

$$ \operatorname{TV}^{\gamma}\biggl( \int_{a}^{\cdot} \bigl[f(s)-f(a) \bigr]\,\mathrm {d}g(s),[a,b]\biggr) \leq2\sum_{k=0}^{+\infty}3^{k} \delta_{k-1}\cdot \operatorname{TV}^{\varepsilon_{k}}\bigl(g,[a,b]\bigr) . $$

#### Proof

We proceed similarly as in the proof of Lemma 2. Define $g_{0}=g$, $f_{0}=f$, $g_{1}:=g_{0}-g_{0}^{\varepsilon_{0}}$, $f_{1}:=f_{0}-f_{0}^{\delta_{0}}$, where $g_{0}^{\varepsilon_{0}}$ is piecewise linear, with possible discontinuities only at the points where *g* is discontinuous, and such that

$$\bigl\Vert g_{0}-g_{0}^{\varepsilon_{0}} \bigr\Vert _{\infty,[a,b]}\leq\varepsilon_{0}\quad\mbox{and}\quad \operatorname{TV}^{0} \bigl(g_{0}^{\varepsilon_{0}},[a,b]\bigr) \le2 \operatorname{TV}^{\varepsilon_{0}/4} \bigl(g_{0},[a,b]\bigr) , $$

and, similarly, $f_{0}^{\delta_{0}}$ is piecewise linear, with possible discontinuities only at the points where *f* is discontinuous, and such that

$$\bigl\Vert f_{0}-f_{0}^{\delta_{0}} \bigr\Vert _{\infty,[a,b]}\leq\delta_{0}\quad\mbox{and}\quad \operatorname{TV}^{0} \bigl(f_{0}^{\delta_{0}},[a,b]\bigr) \le2 \operatorname{TV}^{\delta_{0}/4} \bigl(f_{0},[a,b]\bigr) . $$

For $k=2,3,\ldots$ , $g_{k}:=g_{k-1}-g_{k-1}^{\varepsilon_{k-1}}$, $f_{k}:=f_{k-1}-f_{k-1}^{\delta_{k-1}}$ are defined similarly as $g_{1}$ and $f_{1}$. By the linearity of the Riemann-Stieltjes integral with respect to the integrator, integrating by parts, for $t\in [a,b ]$, $r=1,2,\ldots$ , we have

38 $$\begin{aligned}[b] & \int_{a}^{t} \bigl[f (s )-f (a ) \bigr]\,\mathrm {d}g (s ) \\ &\quad = \int_{a}^{t} \bigl[f_{0} (s )-f_{0} (a ) \bigr]\,\mathrm{d}g^{\varepsilon_{0}} (s )+ \int_{a}^{t} \bigl[f_{0} (s )-f_{0} (a ) \bigr]\,\mathrm{d}g_{1} (s ) \\ &\quad = \int_{a}^{t} \bigl[f_{0} (s )-f_{0} (a ) \bigr]\,\mathrm{d}g_{0}^{\varepsilon_{0}} (s )+ \int_{a}^{t}\,\mathrm{d}f_{0} (s ) \bigl[g_{1} (t )-g_{1} (s ) \bigr] \\ &\quad = \int_{a}^{t} \bigl[f_{0} (s )-f_{0} (a ) \bigr]\,\mathrm{d}g_{0}^{\varepsilon_{0}} (s )+ \int_{a}^{t}\,\mathrm{d}f_{0}^{\delta_{0}} (s ) \bigl[g_{1} (t )-g_{1} (s ) \bigr] \\ &\quad \quad+ \int_{a}^{t}\,\mathrm{d}f_{1} (s ) \bigl[g_{1} (t )-g_{1} (s ) \bigr] \\ &\quad = \int_{a}^{t} \bigl[f_{0} (s )-f_{0} (a ) \bigr]\,\mathrm{d}g_{0}^{\varepsilon_{0}} (s )+ \int_{a}^{t}\,\mathrm{d}f_{0}^{\delta_{0}} (s ) \bigl[g_{1} (t )-g_{1} (s ) \bigr] \\ &\quad \quad+ \int_{a}^{t} \bigl[f_{1} (s )-f_{1} (a ) \bigr]\,\mathrm{d}g_{1} (s )=\cdots \\ &\quad =\sum_{k=0}^{r-1} \biggl( \int_{a}^{t} \bigl[f_{k} (s )-f_{k} (a ) \bigr]\,\mathrm{d}g_{k}^{\varepsilon_{k}} (s )+ \int_{a}^{t}\,\mathrm{d}f_{k}^{\delta_{k}} (s ) \bigl[g_{k+1} (t )-g_{k+1} (s ) \bigr] \biggr) \\ & \quad\quad+ \int_{a}^{t} \bigl[f_{r} (s )-f_{r} (a ) \bigr]\,\mathrm{d}g_{r} (s ). \end{aligned} $$

By Theorem 2 we easily estimate that

39 $$\begin{aligned}[b] & \biggl\Vert \int_{a}^{t} \bigl[f_{r} (s )-f_{r} (a ) \bigr]\,\mathrm{d}g_{r} (s ) \biggr\Vert _{V} \\ & \quad\leq4 \sum_{k=r}^{+\infty}3^{k} \delta_{k-1}\cdot \operatorname{TV}^{\varepsilon_{k}/4}\bigl(g,[a,t ]\bigr) +4\sum _{k=r}^{\infty}3^{k} \varepsilon_{k}\cdot \operatorname{TV}^{\delta_{k}/4}\bigl(f,[a,t]\bigr) \end{aligned} $$

for $r=1,2,\ldots$ . Moreover, for $k=0,1,\ldots$ , similarly as in the proof of Lemma 2, we estimate

40 $$ \biggl\Vert \int_{a}^{t}\,\mathrm{d}f_{k}^{\delta_{k}} (s ) \bigl[g_{k+1} (t )-g_{k+1} (s ) \bigr] \biggr\Vert _{V}\leq2\varepsilon_{k} \operatorname{TV}^{0} \bigl(f_{k}^{\delta_{k}},[a,t]\bigr) \leq2\cdot3^{k} \varepsilon_{k} \operatorname{TV}^{\delta_{k}/4}\bigl(f,[a,t]\bigr) , $$

and

41 $$\begin{aligned}[b] \operatorname{TV}^{0}\biggl( \int_{a}^{\cdot} \bigl[f_{k}(s)-f_{k}(a) \bigr]\,\mathrm {d}g_{k}^{\varepsilon_{k}}(s),[a,b]\biggr) & \leq2 \delta_{k-1} \operatorname{TV}^{0}\bigl(g_{k}^{\varepsilon_{k}},[a,b ]\bigr) \\ & \leq2\cdot3^{k}\delta_{k-1}\cdot \operatorname{TV}^{\varepsilon_{k}/4} \bigl(g,[a,b]\bigr) . \end{aligned} $$

(Notice that, for the function $F_{k}(t):=\int_{a}^{t} [g_{k+1} (t )-g_{k+1} (s ) ]\,\mathrm{d}f_{k}^{\delta_{k}} (s )$, we could not obtain an estimate similar to (41). This is due to the fact that $F_{k}(t_{2})-F_{k}(t_{1})$ cannot be expressed as the integral $\int_{t_{1}}^{t_{2}} [g_{k+1} (t )-g_{k+1} (s ) ]\,\mathrm{d}f_{k}^{\delta_{k}} (s )$.) Defining

$$\gamma(r ):=8\sum_{k=r}^{+\infty}3^{k} \delta_{k-1}\cdot \operatorname{TV}^{\varepsilon_{k}}\bigl(g,[a,b]\bigr) +8\sum _{k=0}^{+\infty }3^{k}\varepsilon _{k}\cdot \operatorname{TV}^{\delta_{k}/4}\bigl(f,[a,b]\bigr) , $$

from (38), (40), and (39) we get

$$\begin{aligned} & \Biggl\Vert \int_{a}^{t} \bigl[f (s )-f (a ) \bigr]\,\mathrm{d}g (s )-\sum_{k=0}^{r-1} \int_{a}^{t} \bigl[f_{k} (s )-f_{k} (a ) \bigr]\,\mathrm{d}g_{k}^{\varepsilon _{k}} (s ) \Biggr\Vert _{V} \\ &\quad \leq\sum_{k=0}^{r-1} \biggl\Vert \int_{a}^{t}\,\mathrm{d}f_{k}^{\delta _{k}} (s ) \bigl[g_{k+1} (t )-g_{k+1} (s ) \bigr] \biggr\Vert _{V}+ \biggl\Vert \int_{a}^{t} \bigl[f_{r} (s )-f_{r} (a ) \bigr]\,\mathrm{d}g_{r} (s ) \biggr\Vert _{V} \\ &\quad \leq\frac{1}{2}\gamma(r ) \end{aligned}$$

for any $t\in[a,b ]$. Let us notice that by the definition of the truncated variation

$$\operatorname{TV}^{\gamma}\biggl( \int_{a}^{\cdot} \bigl[f(s)-f(a) \bigr]\,\mathrm {d}g(s),[a,t]\biggr) $$

is bounded from above by the variation of any function approximating the indefinite integral $\int_{a}^{\cdot} [f (s )-f (a ) ]\,\mathrm {d}g (s )$ with accuracy $\gamma/2$. By this variational property of the truncated variation and by (41) we get

$$\begin{aligned} & \operatorname{TV}^{\gamma(r)}\biggl( \int_{a}^{\cdot} \bigl[f(s)-f(a) \bigr] \,\mathrm{d}g(s),[a,b]\biggr) \\ & \quad\leq \operatorname{TV}^{0}\Biggl(\sum_{k=0}^{r-1} \int_{a}^{\cdot} \bigl[f_{k}(s)-f_{k}(a) \bigr]\,\mathrm{d}g_{k}^{\varepsilon_{k}}(s ),[a,b]\Biggr) \\ & \quad\leq2\sum_{k=0}^{r-1}3^{k} \delta_{k-1}\cdot \operatorname{TV}^{\varepsilon_{k}/4}\bigl(g,[a,b]\bigr) . \end{aligned}$$

Passing to the limit as $r\to+\infty$, we get the assertion. □

Now we are ready to prove Theorem 3.

#### Proof

Let $\gamma>0$. We choose

$$\alpha=\frac{\sqrt{(q-1)(p-1)}+1}{2}, \quad\quad\delta_{-1}=\frac {1}{2}\sup _{a\leq t\leq b} \bigl\Vert f (t )-f (a ) \bigr\Vert _{L (E,V )} $$

and define *β* by the equality

$$2\cdot4^{p} \Biggl(\sum_{k=0}^{+\infty}3^{k+1-p-\alpha(1-\alpha ) (\alpha^{2}/ [ (q-1 ) (p-1 ) ] )^{k}/ (q-1 )} \Biggr) \Vert f \Vert _{p\text{-TV}, [a,b ]}^{p}\delta_{-1}^{1-p} \beta=\gamma. $$

Now, for $k=0,1,\ldots$ , we define

$$\begin{gathered} \delta_{k-1}=3^{- (\alpha^{2}/ [ (q-1 ) (p-1 ) ] )^{k}+1}\delta_{-1}, \\\varepsilon_{k}=3^{- (\alpha^{2}/ [ (q-1 ) (p-1 ) ] )^{k}\alpha/ (q-1 )}\beta. \end{gathered}$$

Using (21), similarly as in the proof of Corollary 1, we estimate

$$\begin{aligned} & \sum_{k=0}^{+\infty}3^{k} \delta_{k-1}\cdot \operatorname{TV}^{\varepsilon_{k}/4}\bigl(g,[a,b ]\bigr) \\ &\quad \leq4^{q-1} \Biggl(\sum_{k=0}^{+\infty }3^{k+1- (1-\alpha) (\alpha^{2}/ [ (q-1 ) (p-1 ) ] )^{k}} \Biggr) \Vert g \Vert _{q\text{-TV}, [a,b ]}^{q}\delta_{-1} \beta^{1-q} \end{aligned}$$

and

$$\begin{aligned} \tilde{\gamma}&: =8\sum_{k=0}^{+\infty}3^{k} \varepsilon_{k}\cdot \operatorname{TV}^{\delta_{k}/4}\bigl(f,[a,b]\bigr) \\ & \leq2\cdot4^{p} \Biggl(\sum_{k=0}^{+\infty}3^{k+1-p-\alpha (1-\alpha) (\alpha^{2}/ [ (q-1 ) (p-1 ) ] )^{k}/ (q-1 )} \Biggr) \Vert f \Vert _{p\text{-TV}, [a,b ]}^{p}\delta_{-1}^{1-p} \beta \\ & =\gamma. \end{aligned}$$

By the monotonicity of the truncated variation, Lemma 3 and the last two estimates we get

$$\begin{aligned} &\operatorname{TV}^{\gamma}\biggl( \int_{a}^{\cdot} \bigl[f(s)-f(a) \bigr]\,\mathrm {d}g(s),[a,b]\biggr) \\&\quad\leq \operatorname{TV}^{\tilde{\gamma}}\biggl( \int_{a}^{\cdot} \bigl[f(s)-f(a) \bigr]\,\mathrm{d}g (s),[a,b]\biggr) \\ &\quad \leq4\sum_{k=0}^{+\infty}3^{k} \delta_{k-1}\cdot \operatorname{TV}^{\varepsilon_{k}}\bigl(g,[a,b ]\bigr) \\ & \quad\leq4^{q} \Biggl(\sum_{k=0}^{+\infty}3^{k+1- (1-\alpha) (\alpha^{2}/ [ (q-1 ) (p-1 ) ] )^{k}} \Biggr) \Vert g \Vert _{q\text{-TV}, [a,b ]}^{q}\delta_{-1} \beta^{1-q} \\ &\quad =\tilde{D}_{p,q} \Vert f \Vert _{p\text{-TV}, [a,b ]}^{pq-p} \Vert f \Vert _{\text{osc}, [a,b ]}^{p+q-pq} \Vert g \Vert _{q\text{-TV}, [a,b ]}^{q}\gamma^{1-q}, \end{aligned}$$

where

$$\begin{aligned} \tilde{D}_{p,q}= {}& 4^{q} \Biggl(\sum _{k=0}^{+\infty}3^{k+1- (1-\alpha ) (\alpha^{2}/ [ (q-1 ) (p-1 ) ] )^{k}} \Biggr) \\ & \times \Biggl(2\cdot4^{p} \Biggl(\sum_{k=0}^{+\infty }3^{k+1-p-\alpha (1-\alpha) (\alpha^{2}/ [ (q-1 ) (p-1 ) ] )^{k}/ (q-1 )} \Biggr) \Biggr)^{q-1}. \end{aligned}$$

From this and the definition of $\Vert \cdot \Vert _{q\text{-TV}, [a,b ]}$ we get

$$\biggl\Vert \int_{a}^{\cdot} \bigl[f (s )-f (a ) \bigr]\,\mathrm{d}g (s ) \biggr\Vert _{q\text{-TV}, [a,b ]}\leq D_{p,q} \Vert f \Vert _{p\text{-TV}, [a,b ]}^{p-p/q} \Vert f \Vert _{\text{osc}, [a,b ]}^{p/q+1-p} \Vert g \Vert _{q\text{-TV}, [a,b ]}, $$

where $D_{p,q}=\tilde{D}_{p,q}^{1/q}$. □
